# Impact of *Sonic Hedgehog*‐dependent sphenoid bone defect on craniofacial growth

**DOI:** 10.1002/cre2.861

**Published:** 2024-04-01

**Authors:** Hélène Guyodo, Aurélie Rizzo, Farah Diab, Fanny Noury, Svetlana Mironov, Marie de Tayrac, Véronique David, Sylvie Odent, Christèle Dubourg, Valérie Dupé

**Affiliations:** ^1^ Univ Rennes, CNRS, IGDR (Institut de Génétique et Développement de Rennes)‐UMR6290 Rennes France; ^2^ Sorbonne Université, Institut National de la Santé et de la Recherche Médicale (INSERM), “Maladies génétiques d'expression pédiatrique” Paris France; ^3^ Faculté des Sciences Pharmaceutiques et Biologiques Univ Rennes, INSERM, LTSI ‐ UMR 1099 Rennes France; ^4^ Service de Génétique Moléculaire et Génomique, CHU Rennes France; ^5^ Service de Génétique Clinique, CHU Rennes France

**Keywords:** basisphenoid bone, hypomorphic mouse model, intersphenoid synchondrosis, sonic hedgehog

## Abstract

**Objectives:**

The main objective of this study was to evaluate how an apparently minor anomaly of the sphenoid bone, observed in a haploinsufficient mouse model for *Sonic Hedgehog* (*Shh*), affects the growth of the adult craniofacial region. This study aims to provide valuable information to orthodontists when making decisions regarding individuals carrying *SHH* mutation.

**Materials and Methods:**

The skulls of embryonic, juvenile and adult mice of two genotypes (*Shh* heterozygous and wild type) were examined and measured using landmark‐based linear dimensions. Additionally, we analysed the clinical characteristics of a group of patients and their relatives with SHH gene mutations.

**Results:**

In the viable *Shh* ^+/^^−^ mouse model, bred on a C57BL/6J background, we noted the presence of a persistent foramen at the midline of the basisphenoid bone. This particular anomaly was attributed to the existence of an ectopic pituitary gland. We discovered that this anomaly led to premature closure of the intrasphenoidal synchondrosis and contributed to craniofacial deformities in adult mice, including a longitudinally shortened skull base. This developmental anomaly is reminiscent of that commonly observed in human holoprosencephaly, a disorder resulting from a deficiency in SHH activity. However, sphenoid morphogenesis is not currently monitored in individuals carrying *SHH* mutations.

**Conclusion:**

Haploinsufficiency of *Shh* leads to isolated craniofacial skeletal hypoplasia in adult mouse. This finding highlights the importance of radiographic monitoring of the skull base in all individuals with SHH gene mutations.

## 1 INTRODUCTION

Many structural birth defects of the craniofacial bones are caused by genetic factors, as the development of the cranial base requires thorough coordination between bone formation, suture closure, and cerebral development (McBratney‐Owen et al., 2008; Wilkie & Morriss‐Kay, 2001). Failure of any one of these processes can result in an abnormally shaped skull and thus functional and esthetic orthodontic issues. The sphenoid and basioccipital bone forms the midline of the skull and has a central role in facial growth (Lieberman et al., 2000). The development of the sphenoid bone is closely linked to that of the pituitary gland, abnormal development of the pituitary leads to changes in the shape of the sphenoid bone (Abele et al., 2014). This occurs in human developmental disorders like holoprosencephaly (HPE), a structural malformation of the forebrain of variable severity. In the most severe form of HPE, the cerebral hemispheres are completely fused. In the mildest form, the only signs of disease are midline defects like a solitary median maxillary central incisor or a cleft palate (Mercier et al., 2011). A sphenoid bone malformation and an abnormal pituitary gland are clinical manifestations of the broad phenotypic spectrum of HPE (Cohen, 2006; Kjær, 2015).

Most cases of HPE are caused by a defect in the Sonic Hedgehog (SHH) signaling pathway (Dubourg et al., 2018; Gregory et al., 2015; Kim et al., 2019). The SHH pathway's functions in early brain development have been extensively described (Crane‐Smith et al., 2021; Mercier et al., 2013). *Shh*‐deficient mouse models show the main clinical signs of HPE, including craniofacial morphological defects (Chiang et al., 1996; Jeong et al., 2004). However, the pathogenesis of these various craniofacial phenotypes has yet to be understood. The present study is the first to describe the phenotype of adult *Shh* ^*+/*^^−^ mutant with a C57BL/6J background. This viable mouse model presents with a unique microform of HPE. We investigated the origin of this defect and its impact on growth of the cranial base and skull. Lastly, we report and comment on the clinical features of a cohort of individuals carrying alterations in the *SHH* gene.

## 2 MATERIAL AND METHODS

### 2.1 Generation of *Shh* ^+/^^−^ mice

The Shhtm1Amc/J mouse strain was purchased from the Jackson Laboratory and maintained on a C57BL/6J background (Janvier Labs). 58 *Shh* ^+/^^−^ and 20 C57Bl/6J mice were crossed to generate animals for experiments

### 2.2 In situ hybridization, histology, and skeletal preparation

Embryo samples were fixed in 4% paraformaldehyde overnight and stored in 100% methanol at −20°C before processing for whole‐embryo in situ RNA hybridization (Ratié et al., 2013). Embryos were embedded in paraffin and eight‐micrometer serial sections were trimmed in the frontal plane and stained with haematoxylin‐eosin reagent. For skeletal preparation, E18.5 embryos and heads of adult animals were processed as described previously (Hamdi‐Rozé et al., 2020). Samples were stained with Alcian blue (Sigma A3157) for cartilage and alizarin red (Sigma A5533) for bone.

### 2.3 Cephalometric analysis

Skulls were imaged with a Nikon AZ100 stereoscope and a DS‐Ri2 camera. All measurements were processed with ImageJ software (NIH). Each value was measured in triplicate. 16 defined landmarks were used to assess the morphology of the base of the E18.5 skull, 34 landmarks were used to delineate the adult skull. Measurements in pixels were converted into millimeters using GraphPad Prism Software. The statistical significance was probed by applying the Kolmogorov‐Smirnov test. Data were expressed as the median ± SD. The threshold for statistical significance was set to *p* < .05 (ns, not significant *p* >.05; *p ≤ .05; **p ≤ .01; ***p ≤ .001; ****p ≤ .0001 vs. the wild type [WT]).

### 2.4 Analysis of the European holoprosencephaly patient cohort

A European HPE network was previously established. Clinical data on 2800 individuals were analysed. Informed consent was obtained from all patients or their legal representatives. The *SHH* gene was sequenced in samples from the probands and their relatives (Dubourg et al., 2018). In this study, 142 individuals carrying an *SHH* gene variant were analysed (70 probands and 72 relatives).

2.5

Human genetics: The protocol was approved by the local independent Health Research ethics committee, University Hospital, France, (reference number: DC‐2015‐2565).

## 3 RESULTS

### 3.1 Pituitary morphogenesis is impaired in *Shh* ^+/^^−^ mice

Homozygous (*Shh* ^*‐*/‐^) mutants are not viable and exhibit severe craniofacial abnormalities; in contrast, heterozygous mutants (*Shh* ^+/^^−^) have previously been described as phenotypically unremarkable (Allen et al., 2007; Chiang et al., 1996). Here, we performed the first comprehensive phenotypic analysis of *Shh* ^+/^^−^ mice with a C57BL/6J background. Unsurprisingly, *Shh* ^+/^^−^ animals and their WT littermates did not differ significantly with regard to survival, fertility and weight (Appendix Figure 1).

**Figure 1:**
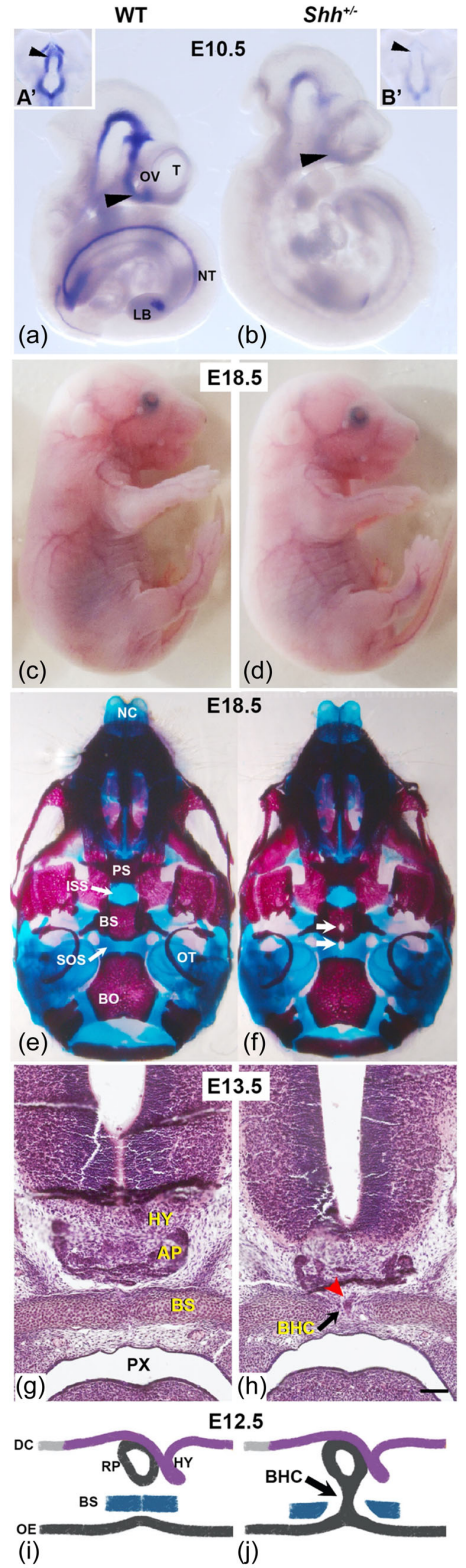
*Shh* ^*+/*^^−^ embryos show a persistent BHC. (a, b) Whole‐embryo in situ RNA hybridization analyses. *Shh* transcripts were detected in WT mice and *Shh* ^*+/*^^−^ mutants at E10.5. (a',b') Ventral view of the dissected forebrain (black arrowhead). (c, d) Lateral view of WT and *Shh* ^*+/*^^−^ embryos at E18.5. (e, f) Ventral views of cranial bone preparations of E18.5 embryos. The mandible and vault have been removed to enable viewing of the basicranium. In (f), the white arrows indicate the persistent of the BHC at the level of the basisphenoid (BS). (g, h) Hematoxylin‐eosin staining of coronal sections from E13.5 heads. Note the foramen on the midline of the BS and the BHC (black arrow). The anterior pituitary gland (AP) descends into the midline space in *Shh* ^*+/*^^−^ embryos (red arrowhead). (i, j) Normal development of the pituitary gland is initiated by an interaction between the hypothalamus (HY) and Rathke's pouch (RP, a derivative of the oral ectoderm [OE]). At E12.5, the BS establishes a definitive barrier between the AP and the OE. In *Shh* ^*+/*^^−^ embryos, an impairment of the pituitary gland's development interferes with closure of the BS bone and results in a BHC (red head arrow). BO, basioccipital; DC, diencephalon; ISS, intersphenoid synchondrosis; LB, limb bud; NC, nasal capsule; NT, neural tube; OT, otic capsule; OV, optic vesicle; PS, presphenoid bone; PX, pharynx; SOS, spheno‐occipital bone; T, telencephalon.

We also studied the expression of *Shh* at E10.5, a stage at which Shh is involved in many developmental processes (Figure 1a,b). *Shh* expression was significantly less intense in *Shh* ^+/^^−^ mice (*n* = 7) than in WT mice (*n* = 8) in all areas that normally express *Shh*. This finding was highlighted in the forebrain region, which gives rise to the ventral hypothalamus (Figure 1a,b). Therefore, deletion of one copy of *Shh* had a strong impact on its mRNA levels in all areas of expression.

To further assess the potential impact of this low level of *Shh* mRNA, we studied the phenotype of E18.5 embryos (Figure 1c–f). There were no external morphological differences between WT mice (*n* = 18) and *Shh* ^+/^^−^ mutants (*n* = 22). As Shh is known to be crucial for skeletal development, (Chiang et al., 1996) we performed a thorough examination of the *Shh* ^+/^^−^ E18.5 skeletons. We observed a fully penetrating opening at the midline of the basisphenoid, which reached the thin cartilaginous bridge of the spheno‐occipital synchondrosis (SOS). E18.5 *Shh* ^+/^^−^ embryos did not show other skeletal alterations described in other models of *Shh* deficiency (Jeong et al., 2004).

Examination of frontal histological sections of the E13.5 *Shh* ^+/^^−^ embryos (*n* = 3) revealed that the basisphenoid was interrupted by an abnormal remnant of epithelium, corresponding to a connection between the anterior pituitary and the oral ectoderm (Figure 1h and Appendix Figure 2). In E13.5 WT embryos, the pituitary gland was found in its usual position on the midline of the basisphenoid bone, which had developed correctly (Figure 1g). The same persistent connection between the pituitary gland and the oral ectoderm was observed at E18.5 (Appendix Figure 2).

The fenestration of the basisphenoid is known as the buccohypophyseal canal (BHC) (Khonsari et al., 2013). In both mice and humans, this canal normally closes during development, and so the link between the pituitary primordium (or Rathke's pouch) and the oral ectoderm disappears. This closure allows the basisphenoid to form a barrier between the pituitary gland and the oral cavity. In the mouse, Rathke's pouch begins to form at E9.5, and the BHC finally closes at E11.5 (Alatzoglou & Dattani, 2009). By E12.5, the sphenoid bone has developed fully and forms a definitive barrier between the pituitary and the ectodermal cavity (Figure 1i,j). Therefore, the persistence of the BHC observed in *Shh* ^+/^^−^ embryos is a result of abnormal pituitary development.

To assess the impact of the persistent Rathke's pouch recess on the morphology of the adult pituitary gland, we examined the brains of adult WT and *Shh* ^+/^^−^ mice. Sagittal and coronal cross‐sections of the MRI images showed that the *Shh* ^+/^^−^ pituitary had small caudal ectopic expansion at the midline of the basisphenoid; this was never observed in WT littermates (Appendix Figure 3). Thus, Shh haploinsufficiency can substantially affect pituitary morphology without causing pituitary dysfunction.

### 3.2 Haploinsufficiency of the *Shh* gene results in the persistence of the BHC at E18.5

**Figure 2:**
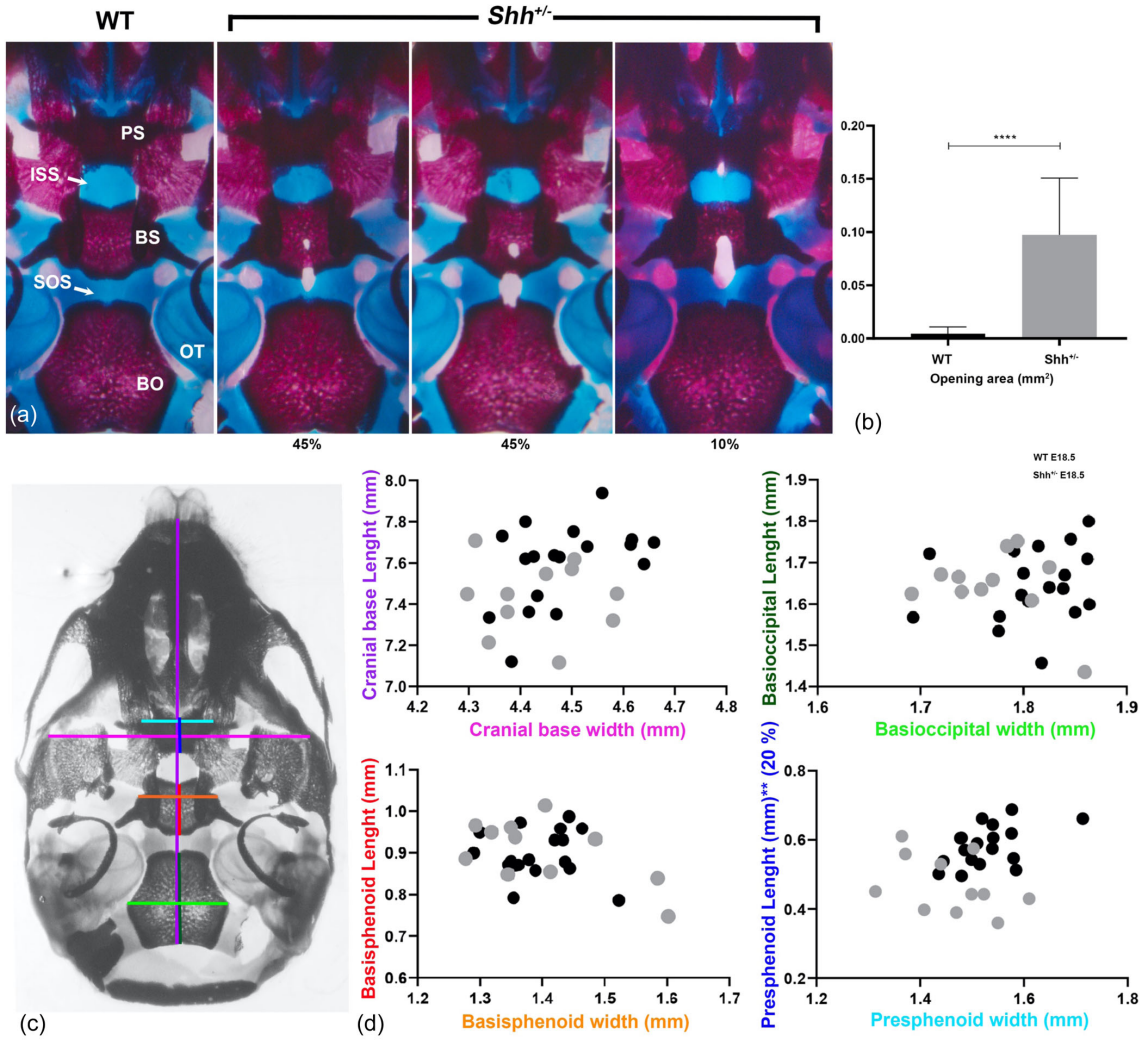
E18.5 *Shh* ^*+/*^^−^ mice display sphenoid bone defects. (a) Ventral views of the basicranium of E18.5 embryos stained with Alizarin red and Alcian blue. (b) Cranial base bone measurements at E18.5. The skeletal elements were measured as indicated by the color bars. These components of the cranial base elements were normally sized in *Shh* ^*+/*^^−^ mice, with the exception of the presphenoid body (PS, 24% shorter; *p* = .0079 vs. the WT). The pink line indicates the most anterolateral point on the alisphenoid; the purple line runs from the most anterior nasal cavity to the posterior basioccipital (BO); the light blue line shows the most anterolateral point of the PS; the dark blue line runs from the most rostral point to the most caudal point of the PS on the midline); the red line runs from the most rostral point to the most caudal point of the basisphenoid (BS) on the midline; the orange line sho‐s the most lateral point of the BS; the light green line shows the most lateral point of the BO; and the dark green line runs from the most rostral point to the most caudal point of the BO on the midline. (c) A histogram representing the area of the opening in *Shh* ^*+/*^^−^. (d). A scatter plot showing the size distribution of the cranial base, BO, BS and PS. There was no reduction of the skull base, other than a shorter median dimension of the PS (the dark blue line). **p ≤ .01; ****p ≤ .0001.

To further assess the size of the opening in the basisphenoid, we compared the WT and *Shh* ^+/^^−^ E18.5 skulls in detail. Although all *Shh* ^+/^^−^ skulls had an opening at the midline of the basisphenoid (*n* = 22), the position and the size of this foramen varied (Figure 2c). This led us to classify the fenestrations into three categories (Figure 2a). The opening either reached the thin cartilage bridge of the SOS (in 45% of the *Shh* ^+/^^−^ skulls) or included the entire SOS up to the basioccipital (45%). The largest openings (10%) have a hole in the posterior midline region of the presphenoid bone, close to the intersphenoid synchondrosis (ISS). This phenotypic variability is probably related to the extent of the physical obstruction of the persistent pituitary epithelial stalk observed in *Shh* ^+/^^−^ embryos (Figure 1). It should be noted that the ISS appeared to be essentially normal in all E18.5 mutants.

We next measured the size of the length and width of the main cranial base bones in 29 E18.5 embryos (WT: *n* = 18; *Shh* ^+/^^−^: *n* = 11) from 4 different littermates (Figure 2b,d). There were no significant differences between *Shh* ^+/^^−^ and WT skulls along the anteroposterior and lateral axes of the base of the skull; and no differences at the level of the basioccipital and basisphenoid bones but the presphenoid was 20% shorter (along the anteroposterior axis) (*p* = .0079, Figure 2d,e).

These observations suggest that at birth, the impact of *Shh* haploinsufficiency on craniofacial skeleton formation was restricted to the sphenoid bones.

### 3.3 Fusion of the intersphenoid synchondrosis in adult *Shh* ^+/^^−^ mice

**Figure 3:**
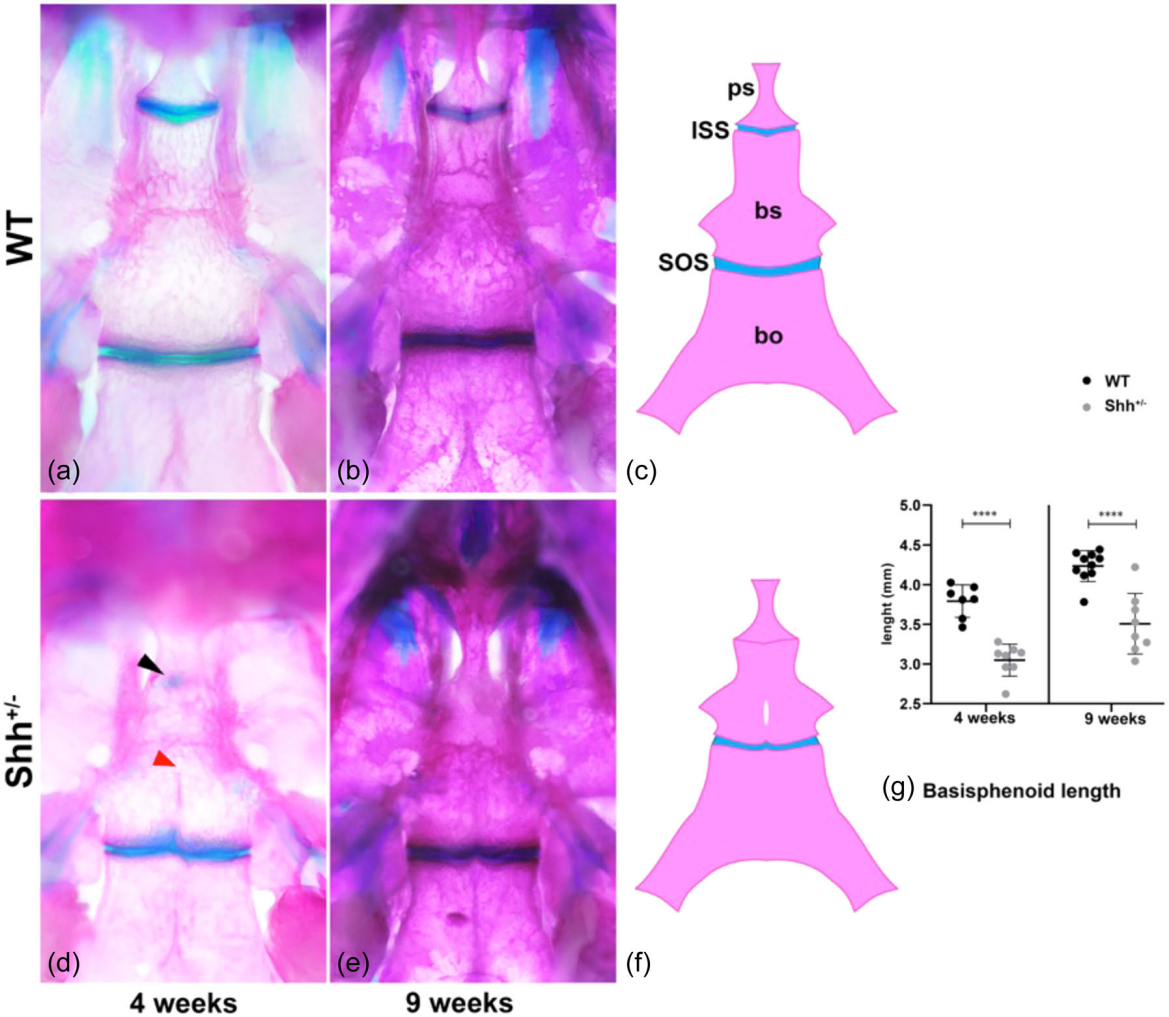
Dysmorphology of the adult cranial base and synchondroses. (a–f) Ventral view of the bones of the skull base in mice at 4 weeks of age (a, b) and 9 weeks of age (c, d), with a schematic illustration of the synchondroses (e, f). The midline of the skull base is formed by the presphenoid (PS), basisphenoid (BS) and basioccipital (BO) bones. The cartilaginous intersphenoid synchondrosis (ISS) and the spheno‐occipital bone (SOS) are located between the mineralized bones. Dissection of the cranial vault facilitated examination of the ventral aspect of the sphenoid bone. The black arrowhead indicates the cartilaginous remnant of the ISS. The red arrowhead indicates an opening on the midline of the BS (G) A scatter diagram showing the size of the basisphenoid bone at 4 weeks and 9 weeks. ****p ≤ .0001.

The *Shh* ^+/‐^ mouse model is thus a unique model for studying the consequences of an isolated sphenoid bone defect on skull growth. We therefore analysed the skull morphology in 4‐week (w)‐old (juvenile) and 9‐w‐old (young adult) *Shh* ^+/^^−^ mice. At both ages, the WT synchondroses were visible as two Alcian‐blue‐stained lines corresponding to cartilage in the ISS (between the basisphenoid and the presphenoid) and the SOS (between the basisphenoid and the basioccipital) (Figure 3a,c,e). In all mutants analysed, the cartilaginous growth plates of the ISS were barely visible remnants at 4‐w (*n* = 15; Figure 3b) and were absent at 9‐w (*n* = 17; Figure 3d,f), whereas the ISS was still not ossified in WT animals. The ectopic position of the remnant ISS (Figure 3b) was due to shortening of the basisphenoid bone, as evidenced by the 20% shorter basisphenoid in *Shh* ^+/^^−^ mice at 4‐w and 9‐w (Figure 3g). A fenestration in the midline of the basisphenoid was still observed in 5 of the 32 *Shh* ^+/^^−^ animals (Figure 3b and Appendix Figure 3F). Furthermore, the SOS appears to be more disorganized in *Shh* ^+/^^−^ mutants than in WT animals, with a variable demarcation between the basisphenoid and the basioccipital (Figure 3d–f). Taken as a whole, these observations showed that the initial anomaly in the basisphenoid leads to premature fusion of the ISS, sphenoid bone hypoplasia, and SOS distortion.

### 3.4 Altered bone growth in *Shh* ^+/^^−^ mice during postnatal craniofacial development

**Figure 4:**
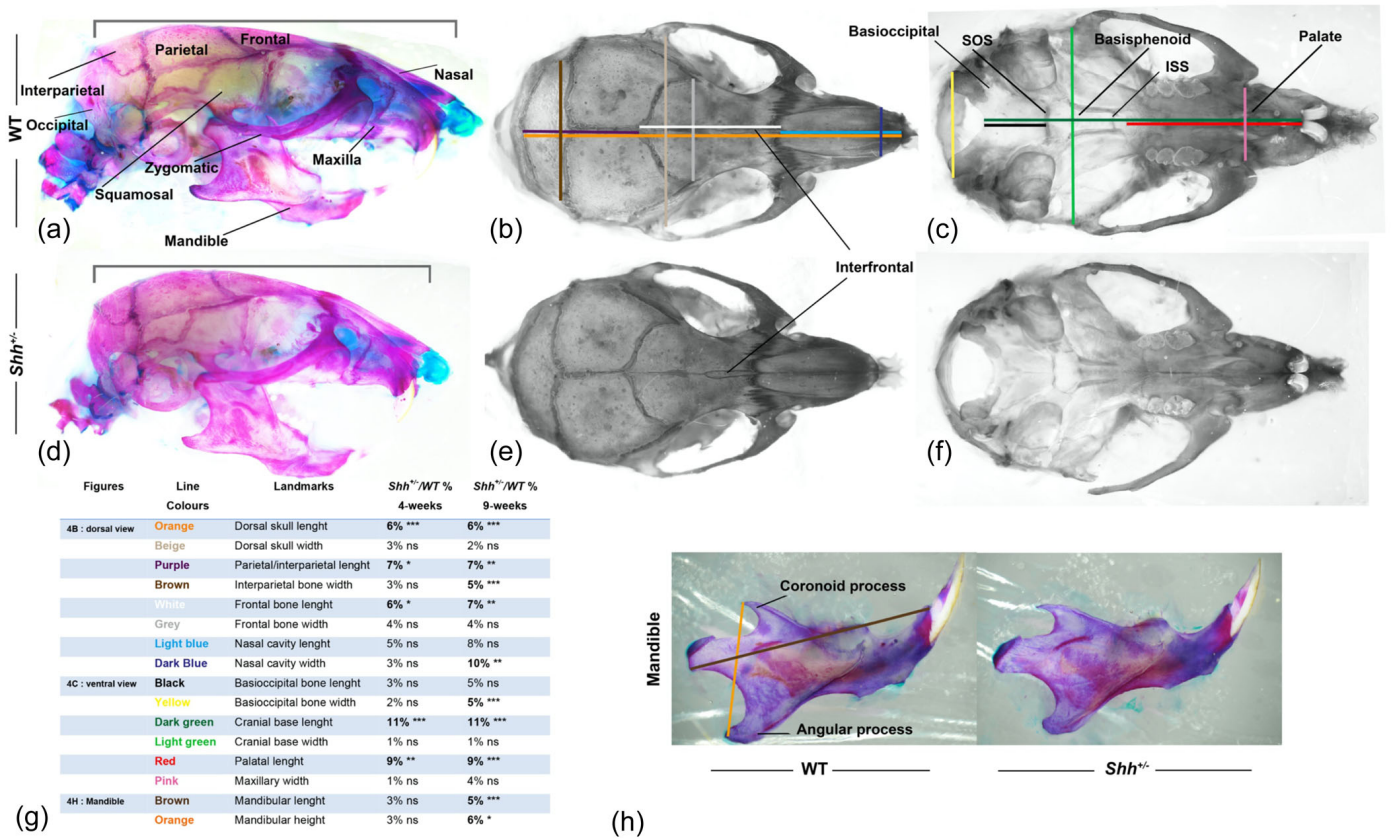
Adult skull skeletons, showing alterations in *Shh* ^*+/*^^−^ mutant mice. Comparison of linear bone measurements in WT vs *Shh* ^*+/*^^−^ skulls at 4 weeks and 9 weeks (a–h). In a lateral view, the bracket is significantly wider in WT than in *Shh* ^+/^^−^ (a–d). Overall dorsal views (b, e) and a ventral view (c, f) of the skull of WT and *Shh* ^*+/*^^−^ mice. The mandibles have been removed to allow viewing of the skull base. (g) Linear bone measurements (by color) for WT and *Shh* ^*+/*^^−^ mice at 4 weeks and 9 weeks. The percentage (%) indicates the reduction in the bone's dimension in *Shh* ^+/^^−^ mice, relative to WT mice. A significant reduction (>5%) in the bone's dimension was determined. ns, not significant; *p ≤ .05; **p ≤ .01; ***p ≤ .001; ****p ≤ .0001 versus the wild type (WT).

To assess the impact of the sphenoid bone defect on skull growth in *Shh* ^+/^^−^ mice, we measured the adult cranial bones. We first noticed that the curvature of the head was more pronounced in *Shh* ^+/^^−^ mice than in WT mice (Figure 4a,d). We measured specific reference points on all the major skull bones in 7 WT and 8 *Shh* ^+/^^−^ mice at 4‐w and in 10 WT and 8 *Shh* ^+/^^−^ mice at 9‐w (Figure 4b,c,e,f,h and Appendix Figure 4). From 4‐w onwards (Figure 4g), the length of the cranial base was significantly shorter in mutants (by 10%) but did not affect the length of the basioccipital. The same difference was observed at adulthood (9‐w), due to a significantly shorter palatal bone (a reduction of 9%: Figure 4c,f,g). In the dorsal view (Figure 4b), the skull measured from the tip of the nasal bone to the back of the skull (the most caudal part of the parietal bone) was slightly but significantly shorter (by 6%) in *Shh* ^+/^^−^ animals at both 4‐w and 9‐w. This was due to the combined reduction in parietal bone length (by 7%) and frontal bone length (by 6%,), whereas nasal bone length was not significantly different. At 4‐w, none of the width measurements were significantly affected. In contrast, the interparietal, nasal and basioccipital bones were significantly narrow (by 5%, 10%, and 5%, respectively) in adult *Shh* ^+/^^−^ mice at 9‐w. Furthermore, growth of the mandible was significantly affected in *Shh* ^+/^^−^ adults at 9‐w: the distance between the coronoid and angular processes of the mandibular bone was 6% shorter in mutants than in WT mice (Figure 4h).

Overall, these results showed that craniofacial growth is impacted in *Shh* ^*+/*^^−^ mice. The initial difference concerned bone length. The severity of the condition increased with age and then affected the bone width.

### 3.5 Clinical features of patients with *SHH* haploinsufficiency

**Figure 5:**
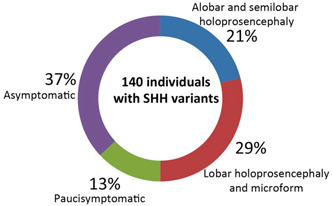
Distribution of the clinical characteristics of 142 individuals from the European HPE cohort bearing a mutation in the *SHH* gene.

The phenotype of the *Shh* ^+/^^−^ mice prompted us to carry out a retrospective analysis of individuals with deleterious *SHH* mutations (Dubourg et al., 2018; Mercier et al., 2011). The European HPE cohort included 70 patients with *SHH* mutations (30% de novo and 70% inherited) and a typical HPE phenotype (Appendix Table 1). All these *SHH* mutations have been classified as pathogenic because they are associated with an HPE phenotype. The phenotype of carriers within a single family can range from alobar HPE (with a single cerebral hemisphere) to a clinically normal state. The most severe cases (29 of the 142 SHH mutation carriers, 20%) led to death (Figure 5). The individuals with less severe forms and microforms (43 out of 142, 30%) did not present typical brain anomalies but did sometimes present ectopic pituitaries, cleft palate, choanal stenosis, or a single median maxillary central incisors. The probands' *SHH* alterations were carried by 72 relatives, who were weakly symptomatic (13%, with microcephaly, intellectual deficiency or hypotelorism) or asymptomatic (37%).

Our retrospective analysis emphasized that in a given family a monoallelic variant of *SHH* may either result in a typical form of HPE or may have no effect (at least with regard to obvious craniofacial anomalies). Unfortunately, we do not have longitudinal CT data on the individuals in the European HPE cohort. It was therefore impossible to assess the impact of *SHH* variants on the basicranium in relatives who had not apparently developed HPE.

## 4 DISCUSSION

Genetic heterogeneity makes it difficult to study the impact of genetics on human craniofacial integrity. In this respect, inbred mouse strains provide genetic standardization and thus experimental reproducibility (Vora, 2017). Phenotype associated with *Shh* inactivation varied based on the mouse strain, with C57BL/6J background showing a more pronounced effect (Lo et al., 2021). Our research discovered that *Shh* ^+/^^−^ mice with a C57BL/6J background exhibit a subtle anterior midline defect. This mouse model therefore mimics specific features of human HPE; i.e. malformation of the pituitary gland and abnormal sphenoid bone.

This developmental anomaly arises from the abnormal persistence of the tissue connecting Rathke's pouch to the oral ectoderm during development. Studies on conditional knockout mice revealed the crucial role of Shh in the ventral diencephalon for Rathke's pouch development (Carreno et al., 2017; Crane‐Smith et al., 2021; Hamdi‐Rozé et al., 2020; Zhao et al., 2012). Recent research also found that mice heterozygous for *Six3* displayed a similar pituitary‐restricted phenotype due to Six3's control of Shh in the ventral forebrain (Bando et al., 2023). Hence, a SHH signaling defect in the ventral diencephalon leads to the persistence of the diverticulum of Rathke's pouch in *Shh* ^+/^^−^. Although *Shh* mRNA levels were abnormally low in E10.5 *Shh* ^+/^^−^ embryos across tissues (Figure 1b), sufficient Shh remained to perform its usual functions during development except controlling Rathke's pouch growth. Hence, forebrain patterning appears to be the most sensitive developmental process affected by Shh deficiency.

Similar anomalies of the sphenoid bones were reported in several mutant mice with early ossification of the ISS‐as early as Day 5 (Bentley‐Ford et al., 2021; Dabovic et al., 2002; Nagata et al., 2011). In *Shh* ^+/^^−^ mice, the ISS closed prematurely while the SOS remained cartilaginous. This premature closure is generally attributed to the accelerated osteogenic differentiation of suture mesenchyme (Funato et al., 2020). However, it has also been suggested that the premature closure of synchondroses is caused by abnormal tensile force resulting from a growth disturbance of cranial base (i.e., hypoplasic sphenoid bones) (Kolpakova‐Hart et al., 2008). This is probably the case in *Shh* ^+/^^−^ mice because ossification of the ISS was caused by the structural defect in the sphenoid bones.

Assessment of defects in the *Shh* ^*+/‐*^ adults revealed that cranial base bones were shorter (relative to the WT) and that the frontal bone exhibited a more pronounced curvature. At 4‐w, only cranial bone lengths were significantly affected in *Shh* ^+/^^−^ mice. However, adult *Shh* ^*+/‐*^ mice also had narrower skull and exacerbated craniofacial anomalies. The premature closure of the ISS is certainly responsible for this skull dysmorphism in *Shh* ^+/^^−^ mice. Previous studies have shown that shortening of the cranial base is responsible for the domed skull phenotype. To accommodate the growing brain volume, the membranous bones of the vault expand outwards and upwards and thus create a domed skull (Parsons et al., 2015). Similarly, premature fusion of the ISS (much as in *Shh* ^+/^^−^) leads to a decrease in the full length of the skull and midface hypoplasia in a mouse model of Apert syndrome bearing an Fgfr2 mutation (Luo et al., 2017).

The coordinated growth of craniofacial bones is crucial for establishing proper spatial relationship between the lower and upper jaws and thus for avoiding malocclusion. In adult *Shh* ^*+/‐*^ mice, the palate was 8% shorter than in WT mice, which is enough to generate malocclusion.

In summary, initial mispatterning in the *Shh* ^+/^^−^ mouse's forebrain induces cranial growth defects but does not significantly affect their viability. However, in humans, such malformation can have a considerable impact on an individual's life. A BHC is reported in 0.42% of the general adult population, and large defects (diameter >1.5 mm) are always associated with craniofacial abnormalities (Abele et al., 2014). We believe that individuals with an *SHH* mutation might have a BHC, which could significantly influence skull and jaw growth in the early years. Although sphenoid bone malformations (including a persistent BHC) have been described in foetuses with HPE (Kjær, 2015) and pituitary hormone deficiencies are present in 54% of living patients, (Traggiai & Stanhope, 2002) routine cranial base CT scans are not typically performed in children with mild forms of HPE. Special attention is paid to craniofacial deformities in children with a clinical diagnosis of HPE but monitoring the skull's growth is not recommended (Mercier et al., 2011; Solomon et al., 2012). Our retrospective study also showed that relatives carrying *SHH* mutations (50% of the cohort) have no apparent clinical manifestations; however, no imaging data on the pituitary and skull base are available. Thus, the accurate radiographic detection of a BHC (using axial cephalography or cone beam CT) might be useful for identifying lesions requiring early orthodontic treatment (Calandrelli et al., 2014).

## 5 CONCLUSION

Taken together, our finding expands the spectrum of anomalies associated with low levels of SHH and has implications for the clinical follow‐up of asymptomatic individuals in families affected by HPE. Clinicians inquire about HPE‐associated mutations when meeting affected families to identify asymptomatic carriers. However, skull growth monitoring is not prescribed unless an obvious facial anomaly is present. Our present findings aim to raise awareness of potential below‐average skull growth in SHH mutation carriers. An in‐depth craniofacial radiographic analysis would be useful for anticipating abnormal cranial growth in young asymptomatic individuals. This analysis becomes essential, as orthodontic intervention for occlusal asymmetry is recommended before the age of 6 (Jaunet et al., 2013). We hope this information will aid orthodontists to make decisions about preventive treatment during follow‐up.

## AUTHOR CONTRIBUTIONS

**H. Guyodo**, **A. Rizzo**, **F. Diab**, **C. Dubourg**, **V. Dupé**: Contributed to conception and design, data acquisition, analysis, and interpretation, drafted and critically revised the manuscript. **F. Noury**, **S. Mironov**, **M. de Tayrac**, **V. David**, **S. Odent**: Data acquisition, data analysis, drafted the manuscript. All authors gave their final approval and agree to be accountable for all aspects of the work. A supplemental appendix to this article is available online. Data can be sent on request.

## CONFLICTS OF INTEREST STATEMENT

No conflict of interest to be declared.

## ETHICS STATEMENT

The local independent Health Research ethics committees approved the protocol. An informed consent was obtained from participating human subjects. All experiments with mouse were approved by the French county veterinary service (Direction Départementale des Services Vétérinaires: APAFiS#27921‐2020102616208630), complied with the European Union guidelines (RL2010/63/EU) and is conformed to the ARRIVE (Animal Research: Reporting of In Vivo Experiments) 2.0 guidelines. Human genetics: The protocol was approved by the local independent Health Research ethics committee, University Hospital, France, (reference number: DC‐2015‐2565).

## Supporting information

Supporting information.

## Data Availability

The data that support the findings of this study are available on request.
