# Supplementary material for: Impact of Sonic Hedgehog‐dependent sphenoid bone defect on craniofacial growth
Source: Clin Exp Dent Res. 2024 Apr 1;10(2):e861. doi: 10.1002/cre2.861 (PMC10982674; doi:10.1002/cre2.861)
Supplement: Supplementary file 1 — Supporting information. [file CRE2-10-e861-s001.pdf]

Appendix files:

Impact of *Sonic Hedgehog*-dependent Sphenoid Bone Defect on Craniofacial Growth

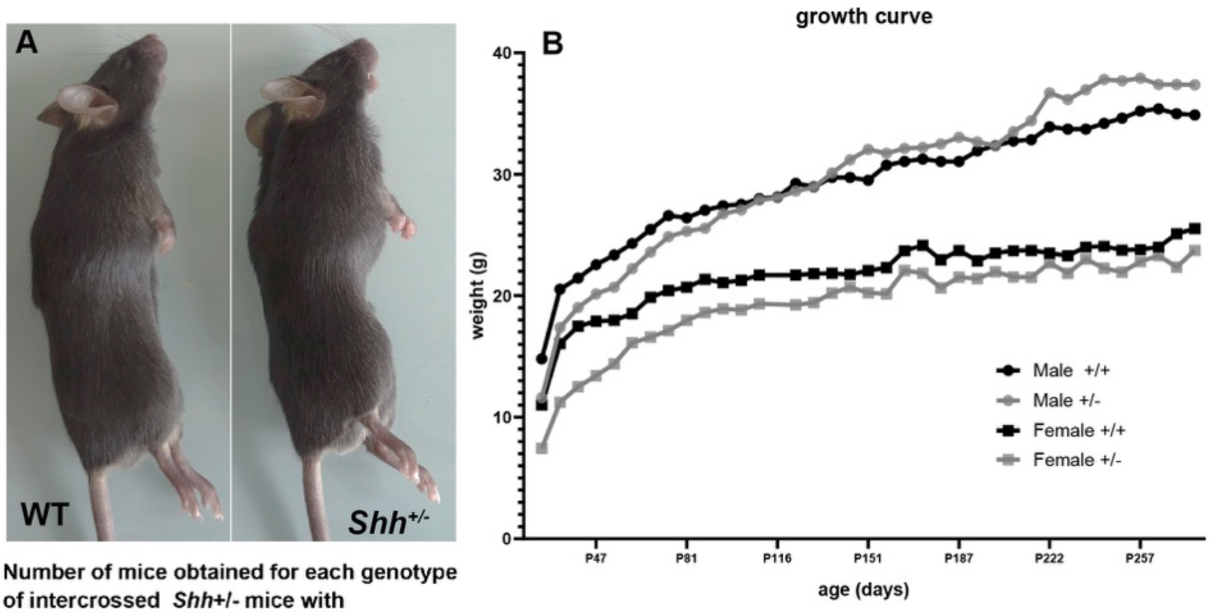

Number of mice obtained for each genotype of intercrossed *Shh+/-* mice with WT

Stages	Genotypes (% expected)* Shh+/+ (50%)	Shh+/- (50%)
E18.5	32 (54%)	27 (46%)
Newborn	17 (43%)	23 (57%)

* derived from expected Mendelian ratios

C

Cross between 22 *Shh+/-* females and WT males

number of females collected	22
number of embryos per female	7,1

Cross between 19 Shh+/- males and WT females

number of females collected	19
number of embryos per female	6,8

D

Appendix Figure 1: ***Shh*$^{+/-}$ adult mice are fertile and grow normally:** A: male mice at 9 w of age. B: body weight of male and female *Shh*$^{+/-}$ mice vs WT littermates, measured weekly for the first 12 months of life. C: the proportion of *Shh*$^{+/-}$ newborns obtained is close to that expected. D: fertility tests conducted on male and female *Shh*$^{+/-}$ animals, showing the number of embryos obtained from each cross.

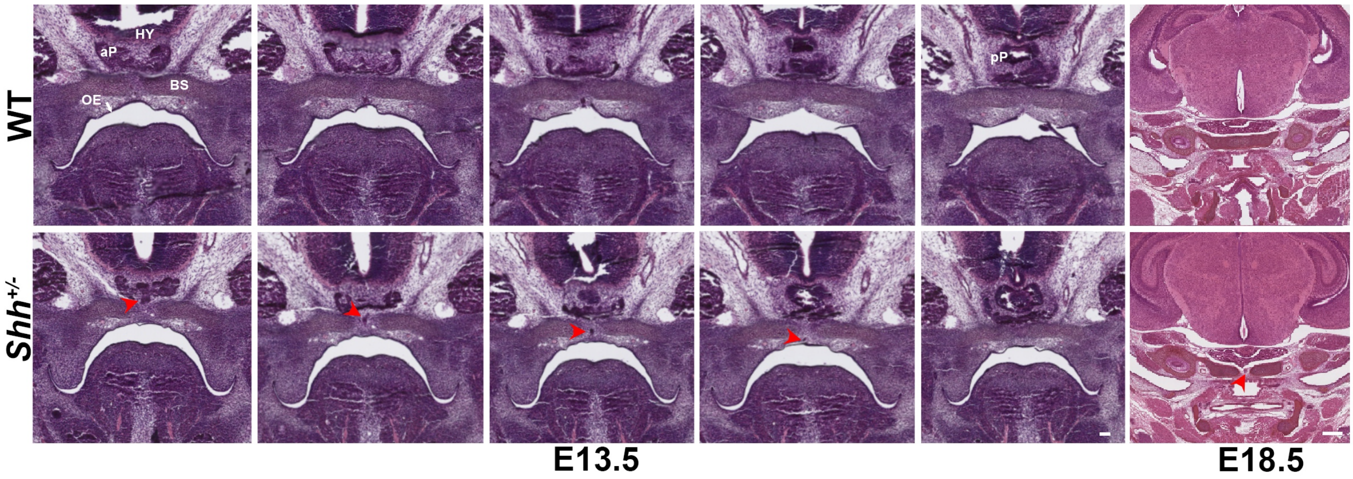

Appendix Figure 2: Haematoxylin-eosin staining of serial frontal sections of WT and *Shh*$^{+/-}$embryos at E13.5 along the anteroposterior axis, and histological slices of the WT and *Shh*$^{+/-}$embryos at E18.5. Red arrowheads indicate the pituitary gland connected to the oral ectoderm (OE). aP, anterior pituitary; BS, basisphenoid bone; HY, hypothalamus; pP, posterior pituitary. Scale bar = 200μM

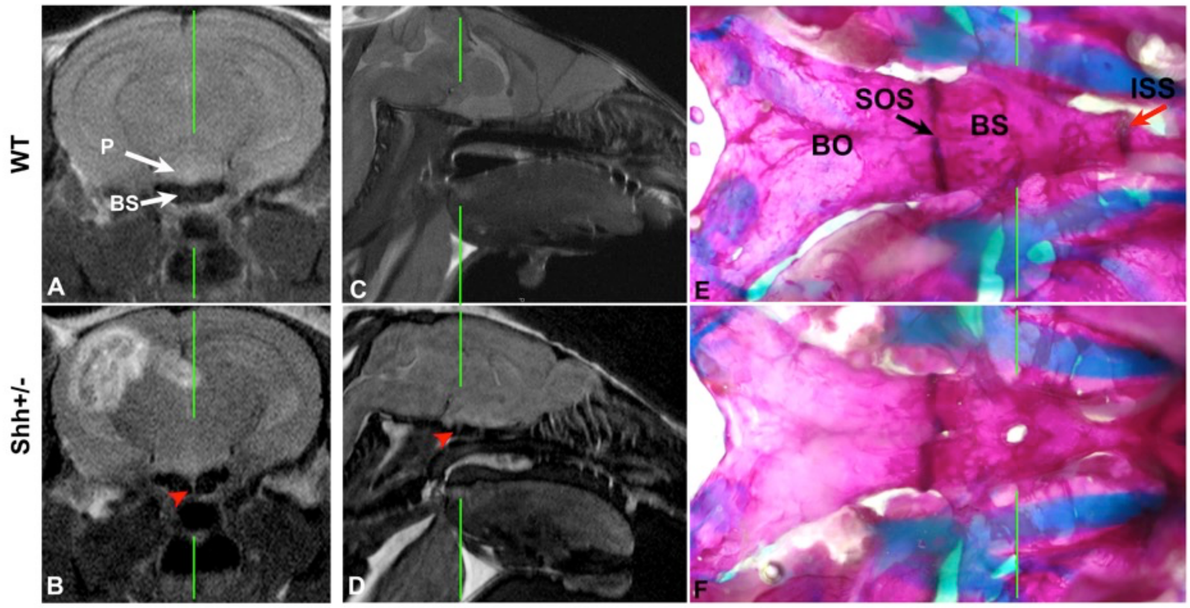

Appendix Figure 3: (A-D) Brain T2w axial (A,B) and sagittal (C,D) images of one adult of *Shh*$^{+/-}$ group (B,D) and one adult of the WT group (A,C). These MRI images (T2-weighted) reveal structural abnormalities in *Shh*$^{+/-}$ adult mice (red arrow in B,D). (E,F) corresponding ventral view of the skull preparation stained with Alcian and alizarin red. Green lines correspond to the position of the buccohypophyseal canal. BO, basioccipital; BS, basisphenoid; ISS, intersphenoid synchondrosis; P: pituitary; SOS, spheno-occipital bone.

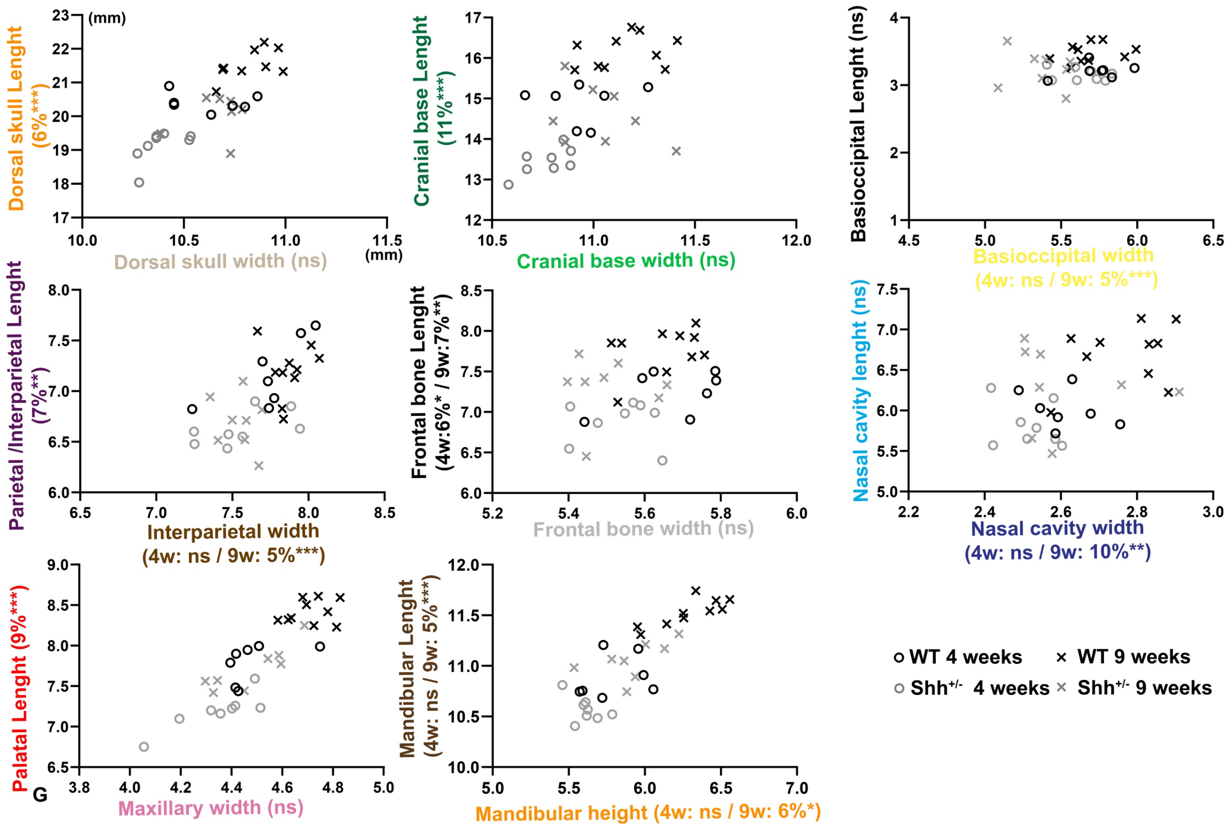

Appendix Figure 4: Comparison of linear distances in WT vs. *Shh*$^{+/-}$ adult skulls. Significance was determined in the Kolmogorov-Smirnov *test.* Bone reductions are indicated in bold. ns = not significant; * *P* ≤ 0.05; ** *P* ≤ 0.01; *** *P* ≤ 0.001; **** *P* ≤ 0.0001 versus the wild type (WT).

Exon	SHH Mutations	Predicted protein change	Inheritance	Relatives with mutation and without HPE
Exon 1	c.17G>C	p.Arg6Thr	Maternal	1
	c.17G>C	p.Arg6Thr	Maternal	1
	c.50T>C	p.Leu17Pro	Maternal	1
	c.57C>A	p.Cys19X	Familial	1
	c.72C>A	p.Cys24X	Familial	3
	c.87delG	p.Phe30Serfs*10	Familial	1
	c.119_120delCC	p.Pro41PhefsX22	De novo	
	c.154G>A	p.Ala52Thr	Maternal	1
	c.157G>A	p.Glu53Lys	Familial	1
	c.201delC	p.Arg68Glufs*30	De novo	
	c.211delG	Frameshift	Paternal	4
	c.248A>T	p.Asp83Val	Paternal	1
	c.298C>T	p.Gln100X	De novo	
	c.300G>C	p.Gln100His	De novo	
Exon 2	c.305G>A	p.Cys102Tyr	Paternal	1
	c.316_321delTTGAAC	p.Leu106_Asn107del	De novo	
	c.329C>A	p.Ala110Asp	Maternal	4
	c.349T>C	p.Trp1171Arg	Maternal	2
	c.388G>T	p.Glu130X	Paternal	1
	c.404C>G	p.Ser135X	De novo	
	c.423C>A	p.Tyr141*	De novo	
	c.428G>A	p.Gly143Asp	Maternal	1
	c.431G>T	p.Arg144Leu	Paternal	1
	c.439G>A	p.Asp147Asn	De novo	
	c.449C>G	p.Thr150Arg	De novo	
	c.449C>A	p.Thr150Lys	De novo	
	c.469A>T	p.Lys157X	Paternal	1
	c.474C>G	p.Tyr158X	Maternal	3
	c.494C>T	p.Ala165Val	De novo	
	c.503C>G	p.Ala168Gly	Maternal	1
	c.511G>C	p.Asp171His	Maternal	1
	c.521A>G	p.Tyr174Cys	Paternal	1
	c.526_534del	p.Glu176_Lys178del	Maternal	1
	c.551C>T	p.Ser184Leu	Paternal	1
	c.554T>C	p.Val185Gly	De novo	
	c.562G>C	p.Glu188Gln	Maternal	1
	c.587delG	p.Gly196GlufsX20	De novo	
	c.587G>A	p.Gly196Glu	Maternal	1
	c.592T>A	p.Cys198Ser	Paternal	1
	c.602G>A	p.Gly201Asp	Familial	1
Exon 3	c.653T>C	p.Leu218Arg	De novo	
	c.664G>A	p.Asp222Asn	Maternal	4
	c.674T>C	p.Leu225Pro	Paternal	1
	c.677C>A	p.(Ala226Glu)	Maternal	1
	c.698T>G	p.Leu233Arg	Paternal	1
	c.701T>C	p.(Leu234Pro)	Maternal	1
	c.707G>A	p.Ser236Asn	Maternal	1
	c.708C>A	p.Ser236Arg	De novo	
	c.721T>C	p.Phe241Val	Maternal	1
	c.730del	p.Arg244Alafs*11	De novo	
	c.812T>C	p.Leu271Pro	Maternal	2
	c.281_282dup	p.Asp95ArgfsX4	De novo	
	c.928C>T	p.Arg310Cys	Familial	1
	c.980T>C	p.Leu327Pro	Familial	1
	c.995T>C	p.Val332Ala	De novo	
	c.995T>C	p.Val332Ala	Familial	1
	c.1015G>T	p.Glu339X	Familial	1
	c.1040C>A	p.Pro347Gln	Paternal	4
	c.1040C>T	p.Pro347Leu	Familial	1
	c.1040C>G	p.Pro347Arg	Paternal	2
	c.1055G>A	p.Gly352Asp	De novo	
	c.1061T>C	p.Ile354Thr	De novo	
	c.1067_1069del	p.Ile356del	De novo	
	c.1085C>A	p.Ser362X	Paternal	1
	c.1091A>G	p.Tyr364Cys	Maternal	1
	c.1091A>G	p.Tyr364Cys	Maternal	1
	c.1097	p.Glu368*	Maternal	5
	c.1142G>C	p.Arg381Pro	Maternal	1
	c.1142G>C	p.Arg381Pro	Paternal	1
	c.1157_1180del	p.Leu386_Ala393del	Paternal	1

Appendix Table 1: List of *SHH* gene alterations in the European HPE cohort.

Supplementary Material and methods:

MRI data acquisition and analysis:

Adult (12-month-old) mice were imaged at the ARCHE animal facility in Rennes (France). *In vivo* brain images were acquired at 4.7 T using an MRI device dedicated to small animal imaging (Bruker BioSpec 47/40 USR), equipped with a BG-06 gradient system (400 mT.m-1), a birdcage coil (36 mm inner diameter) and ParaVision 5.1 software (Bruker Biospin MRI, Wissembourg, France). The acquisition protocol included anatomical T2-weighted images (T2w) in axial and sagittal orientations. A fast and multislice spin echo sequence (rapid acquisition with relaxation enhancement) was used with a repetition time of 2400 ms, an effective echo time of 30 ms and an acceleration factor of 8. For axial images the acquisition parameters were: 2D mode; field of view of 25.6x32.0 mm2; matrix of 256x320; in-plane spatial resolution of 100x100 µm2; 17 slices of 500 µm in thickness; 32 averages; acquisition time of 28 min. For sagittal images the acquisition parameters were: 3D mode; field of view of 19.2x19.2 mm2; 32 slices of 750 µm in thickness; matrix of 256x256; in-plane spatial resolution of 75x75 µm2; 6 averages; acquisition time of 32 min. All T2w images were analyzed using Horos software (version 2.2.0, horosproject.org; ExploreDTI, version 4.8.6, Utrecht).
